# The genome sequence of a beetle-killing wasp, *Tiphia femorata* (Fabricius, 1775)

**DOI:** 10.12688/wellcomeopenres.18893.1

**Published:** 2023-02-03

**Authors:** Liam M. Crowley, University of Oxford and Wytham Woods Genome Acquisition Lab, Darwin Tree of Life Barcoding collective, Wellcome Sanger Institute Tree of Life programme, Wellcome Sanger Institute Scientific Operations: DNA Pipelines collective, Tree of Life Core Informatics collective, Damon-Lee Pointon, Darwin Tree of Life Consortium

**Affiliations:** 1 Department of Biology, University of Oxford, Oxford, Oxfordshire, UK; 2 Wellcome Sanger Institute, Hinxton, Cambridgeshire, UK

**Keywords:** Tiphia femorata, beetle killing wasp, genome sequence, chromosomal, Hymenoptera

## Abstract

We present a genome assembly from an individual female *Tiphia femorata* (a beetle-killing wasp; Arthropoda; Insecta; Hymenoptera; Tiphilidae). The genome sequence is 276 megabases in span. Most of the assembly (98.73%) is scaffolded into 12 chromosomal pseudomolecules. The complete mitochondrial genome was also assembled and is 22.4 kilobases in length. Annotation of the genome in Ensembl identified 10,470 protein-coding genes.

## Species taxonomy

Eukaryota; Metazoa; Ecdysozoa; Arthropoda; Hexapoda; Insecta; Pterygota; Neoptera; Endopterygota; Hymenoptera; Apocrita; Aculeata; Tiphioidea; Tiphiidae; Tiphiinae; *Tiphia*; *Tiphia femorata* (Fabricius, 1775) (NCBI:txid330862).

## Background

*Tiphia femorata* (Fabricius, 1775), a beetle-killing wasp, is an abundant species in most of Europe, the eastern Palaearctic and North Africa ( Museum für Naturkunde, no date; Peeters, 2004). In Great Britain, it mainly inhabits warmer, dry and semi-arid grasslands and meadows, and has been recorded from Cornwall to Kent, south Wales and north to Norfolk, and has also been sighted in the Channel Islands ( Archer, 2001). *T. femorata* is a univoltine species and can be encountered from June through September, feeding on nectar and pollen of flowers (especially on *Apiaceae* species).

*Tiphia femorata* is a small wasp: lengths range from 5 to 12 mm in males and from 6 to 15 mm in females. The body is entirely black, except for the rear two pairs of legs, which are reddish brown ( Museum für Naturkunde, no date).

*Tiphia femorata* is the most common species of Tiphiidae. Like most members of Tiphiidae, it is a parasitoid. The larvae of *T. femorata* prey on the Scarabaeidae *Rhizotrogus solstitialis* and *Anisoplia austriaca* and several *Aphodius* species (Aphodiidae) ( Bogusch, 2007). The female burrows into the soil to find the beetle larval host, stinging the victim to temporarily paralyse it, then laying an egg in the cuticle of the host. The *T. femorata* larva hatches and feed externally on the beetle grub.

The genome of *T. femorata* was sequenced as part of the Darwin Tree of Life Project, a collaborative effort to sequence all named eukaryotic species in the Atlantic Archipelago of Britain and Ireland. Here we present a chromosomally complete genome sequence for *T. femorata*, based on one female specimen from Wytham Woods, Oxfordshire, UK.

### Genome sequence report

**Figure 1.**
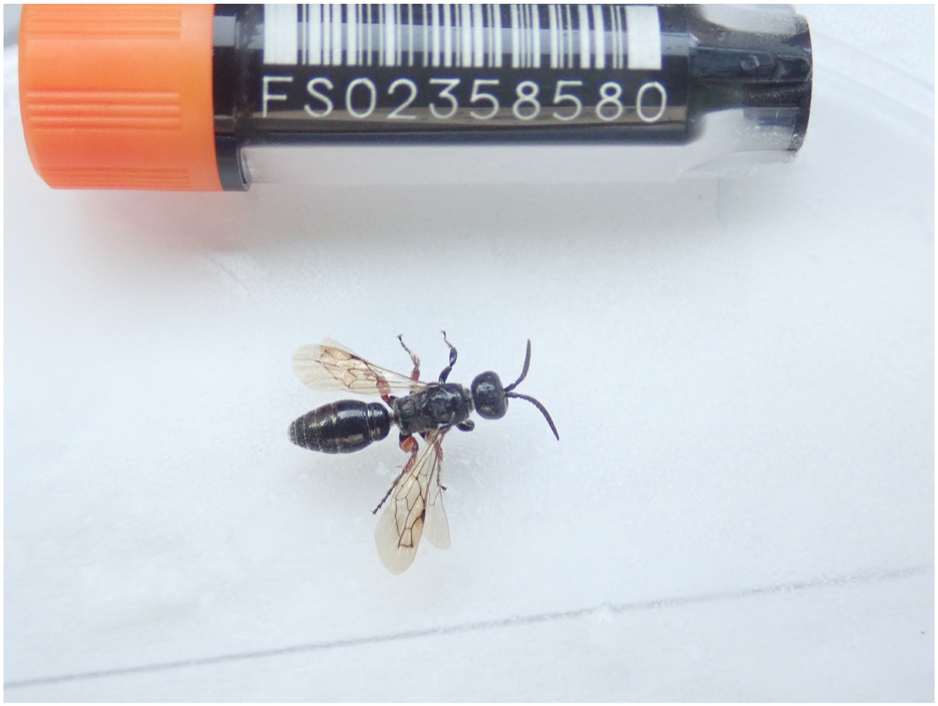
Image of the *Tiphia femorata* specimen taken prior to preservation and processing.

The genome was sequenced from a single female *T. femorata* specimen ( Figure 1) collected in Wytham Great Wood. A total of 75-fold coverage in Pacific Biosciences single-molecule HiFi long reads was generated. Primary assembly contigs were scaffolded with chromosome conformation Hi-C data. Manual assembly curation corrected 11 missing joins or misjoins and removed 1 haplotypic duplication, reducing the assembly size by 0.18% and the scaffold number by 5.66%, and increasing the scaffold N50 by 6.04%.

**Figure 2.**
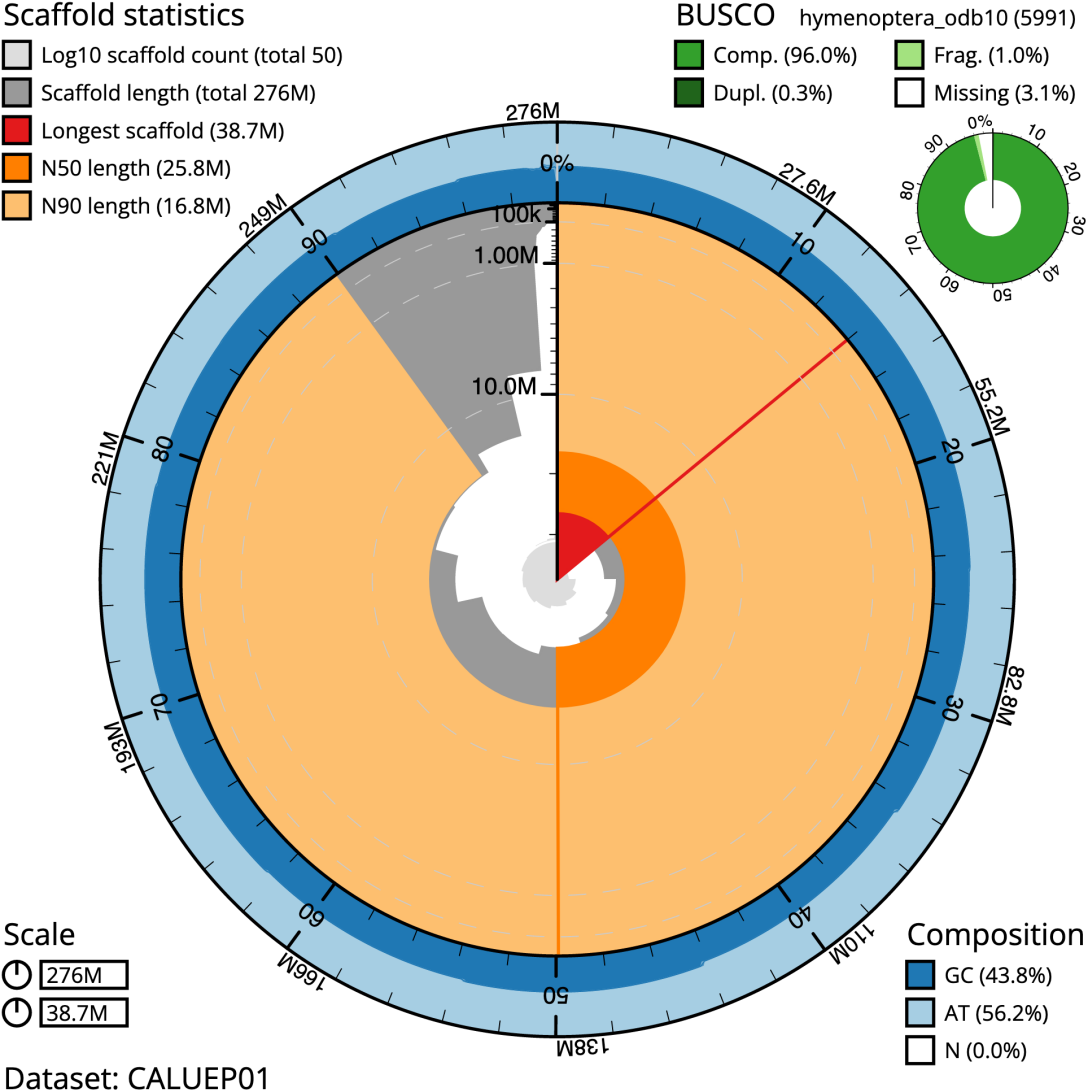
Genome assembly of *Tiphia femorata*, iyTipFemo1.1: metrics. The BlobToolKit Snailplot shows N50 metrics and BUSCO gene completeness. The main plot is divided into 1,000 size-ordered bins around the circumference with each bin representing 0.1% of the 276,162,441 bp assembly. The distribution of scaffold lengths is shown in dark grey with the plot radius scaled to the longest scaffold present in the assembly (38,663,132 bp, shown in red). Orange and pale-orange arcs show the N50 and N90 scaffold lengths (25,801,794 and 16,818,620 bp), respectively. The pale grey spiral shows the cumulative scaffold count on a log scale with white scale lines showing successive orders of magnitude. The blue and pale-blue area around the outside of the plot shows the distribution of GC, AT and N percentages in the same bins as the inner plot. A summary of complete, fragmented, duplicated and missing BUSCO genes in the hymenoptera_odb10 set is shown at the top right. An interactive version of this figure is available at https://blobtoolkit.genomehubs.org/view/iyTipFemo1.1/dataset/CALUEP01/snail.

The final assembly has a total length of 276 Mb in 50 sequence scaffolds with a scaffold N50 of 25.8 Mb ( Table 1). Most (98.73%) of the assembly sequence was assigned to 12 chromosomal-level scaffolds, representing 12 autosomes (numbered by sequence length) ( Figure 2– Figure 5; Table 2). The assembly has a BUSCO v5.3.2 ( Manni *et al.*, 2021) completeness of 96.0% (single 95.7%, duplicated 0.3%) using the OrthoDB v10 hymenoptera reference set ( *n* = 5,991). While not fully phased, the assembly deposited is of one haplotype. Contigs corresponding to the second haplotype have also been deposited.

**Table 1.** Genome data for *Tiphia femorata*, iyTipFemo1.1.

*Project accession data*	
Assembly identifier	iyTipFemo1.1
Species	*Tiphia femorata*
Specimen	iyTipFemo1 (genome assembly, Hi-C)
NCBI taxonomy ID	330862
BioProject	PRJEB51917
BioSample ID	SAMEA7520499
Isolate information	Female, whole organism (iyTipFemo1)
*Raw data accessions*	
PacificBiosciences SEQUEL II	ERR9467445
Hi-C Illumina	ERR9435037
*Genome assembly*	
Assembly accession	GCA_944319695.1
*Accession of alternate haplotype*	GCA_944319705.1
Span (Mb)	276.2
Number of contigs	76
Contig N50 length (Mb)	12.7
Number of scaffolds	50
Scaffold N50 length (Mb)	25.8
Longest scaffold (Mb)	38.7
BUSCO * genome score	C:96.0%[S:95.7%,D:0.3%], F:1.0%,M:3.1%,n:5,991
Genome annotation	
Number of protein-coding genes	10,470
Number of non-coding genes	1,219
Number of gene transcripts	18,538

*BUSCO scores based on the hymenoptera_odb10 BUSCO set using v5.3.2. C = complete [S = single copy, D = duplicated], F = fragmented, M = missing, n = number of orthologues in comparison. A full set of BUSCO scores is available at https://blobtoolkit.genomehubs.org/view/iyTipFemo1.1/dataset/CALUEP01/busco.

**Figure 3.**
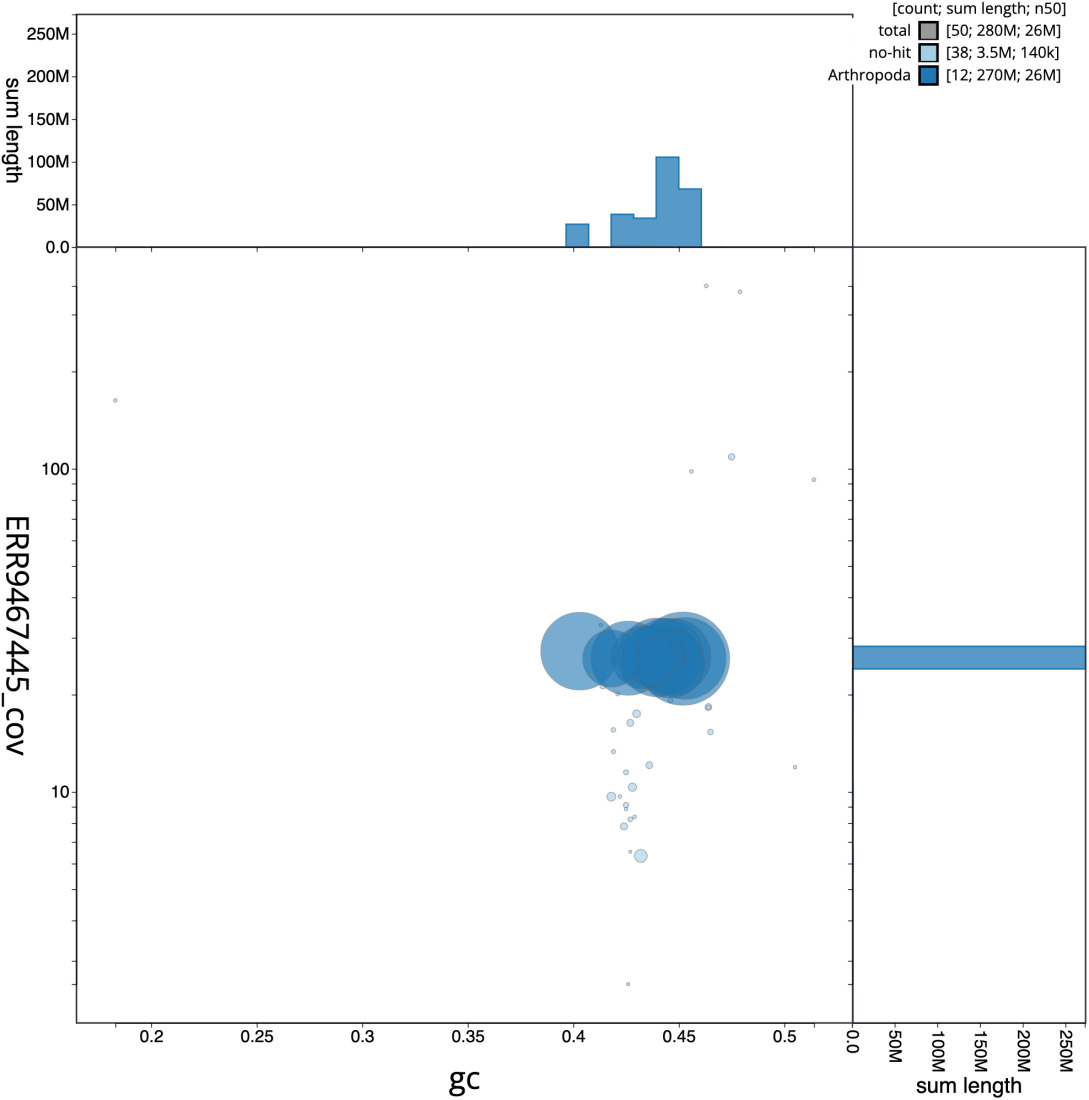
Genome assembly of *Tiphia femorata*, iyTipFemo1.1: GC coverage. BlobToolKit GC-coverage plot. Scaffolds are coloured by phylum. Circles are sized in proportion to scaffold length. Histograms show the distribution of scaffold length sum along each axis. An interactive version of this figure is available at https://blobtoolkit.genomehubs.org/view/iyTipFemo1.1/dataset/CALUEP01/blob.

**Figure 4.**
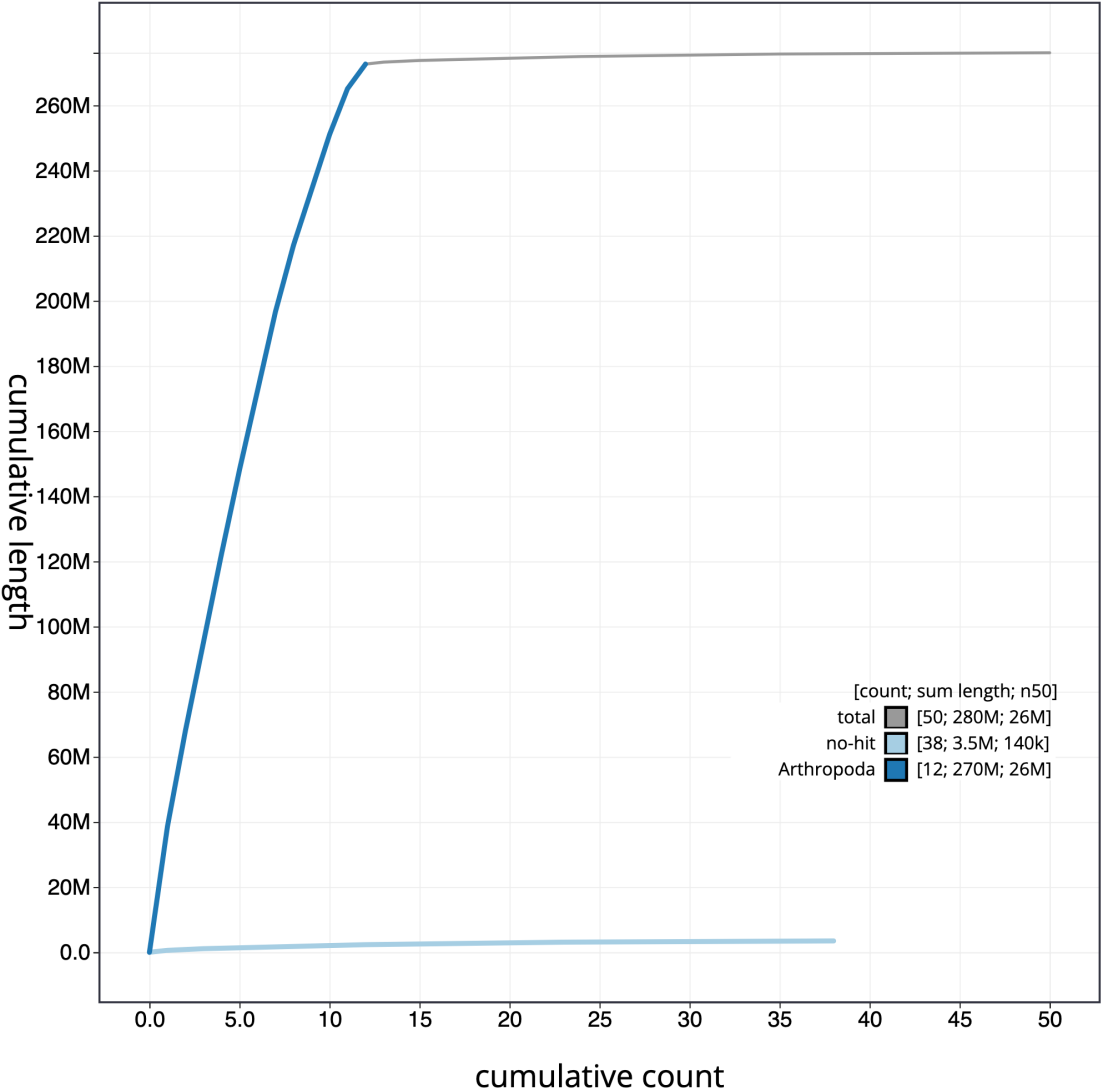
Genome assembly of *Tiphia femorata*, iyTipFemo1.1: cumulative sequence. BlobToolKit cumulative sequence plot. The grey line shows cumulative length for all scaffolds. Coloured lines show cumulative lengths of scaffolds assigned to each phylum using the buscogenes taxrule. An interactive version of this figure is available at https://blobtoolkit.genomehubs.org/view/iyTipFemo1.1/dataset/CALUEP01/cumulative.

**Figure 5.**
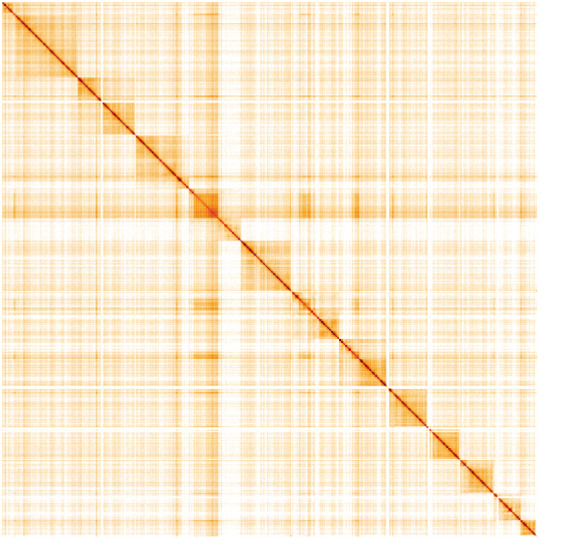
Genome assembly of *Tiphia femorata*, iyTipFemo1.1: Hi-C contact map. Hi-C contact map of the iyTipFemo1.1 assembly, visualised in HiGlass. Chromosomes are arranged in size order from left to right and top to bottom. The interactive Hi-C map can be viewed at https://genome-note-higlass.tol.sanger.ac.uk/l/?d=eT742vyJSUO1PVEDGgOgZg.

**Table 2.** Chromosomal pseudomolecules in the genome assembly of *Tiphia femorata*, iyTipFemo1.1.

INSDC accession	Chromosome	Size (Mb)	GC%
OX090895.1	1	38.66	45.2
OX090896.1	2	29.45	45.3
OX090897.1	3	27.37	44.0
OX090898.1	4	26.79	40.3
OX090899.1	5	25.8	44.7
OX090900.1	6	24.33	42.6
OX090901.1	7	24.28	44.4
OX090902.1	8	20.4	44.6
OX090903.1	9	17.09	43.3
OX090904.1	10	16.82	43.8
OX090905.1	11	14.14	41.8
OX090906.1	12	7.55	44
OX090907.1	MT	0.02	18.5

### Genome annotation report

The *T. femorata* iyTipFemo1.1 genome assembly was annotated using the Ensembl rapid annotation pipeline ( Table 1; https://rapid.ensembl.org/Tiphia_femorata_GCA_944319695.1/). The resulting annotation includes 18,538 transcribed mRNAs from 10,470 protein-coding and 1,219 non-coding genes.

## Methods

### Sample acquisition and nucleic acid extraction

A single female *T. femorata* specimen (iyTipFemo1) was collected on 13 August 2019 in Wytham Great Wood, Oxfordshire (biological vice-county: Berkshire), UK (latitude 51.773, longitude –1.333). The specimen was netted by Liam Crowley (University of Oxford), who also identified the species. The specimen was then snap-frozen on dry ice.

DNA was extracted at the Tree of Life laboratory, Wellcome Sanger Institute. The iyTipFemo1 sample was weighed and dissected on dry ice with tissue set aside for Hi-C sequencing. Whole organism tissue was disrupted using a Nippi Powermasher fitted with a BioMasher pestle. High molecular weight (HMW) DNA was extracted using the Qiagen MagAttract HMW DNA extraction kit. HMW DNA was sheared into an average fragment size between 12–20 kb in a Megaruptor 3 system with a speed setting of 30. Sheared DNA was purified by solid-phase reversible immobilisation using AMPure PB beads with a 1.8X ratio of beads to sample to remove the shorter fragments and concentrate the DNA sample. The concentration of the sheared and purified DNA was assessed using a Nanodrop spectrophotometer and Qubit Fluorometer and Qubit dsDNA High Sensitivity Assay kit. Fragment size distribution was evaluated by running the sample on the FemtoPulse system.

### Sequencing

Pacific Biosciences HiFi circular consensus sequencing libraries were constructed according to the manufacturers’ instructions. Sequencing was performed by the Scientific Operations core at the Wellcome Sanger Institute on a Pacific Biosciences SEQUEL II (HiFi) instrument. Hi-C data were generated in the Tree of Life laboratory from the remaining whole organism tissue of iyTipFemo1 using the Arima v2 kit and sequenced on a HiSeq 10X instrument. 

### Genome assembly

Assembly was carried out with Hifiasm ( Cheng *et al.*, 2021) and haplotypic duplication was identified and removed with purge_dups ( Guan *et al.*, 2020). The assembly was then scaffolded with Hi-C data ( Rao *et al.*, 2014) using YaHS ( Zhou *et al.*, 2022). The assembly was checked for contamination and corrected using the gEVAL system ( Chow *et al.*, 2016) as described previously ( Howe *et al.*, 2021). Manual curation ( Howe *et al.*, 2021) was performed using gEVAL, HiGlass ( Kerpedjiev *et al.*, 2018) and Pretext ( Harry, 2022). The mitochondrial genome was assembled using MitoHiFi ( Uliano-Silva *et al.*, 2021), which performs annotation using MitoFinder ( Allio *et al.*, 2020). The genome was analysed and BUSCO scores were generated within the BlobToolKit environment ( Challis *et al.*, 2020). Table 3 contains a list of all software tool versions used, where appropriate.

**Table 3.** Software tools used.

Software tool	Version	Source
BlobToolKit	3.4.0	Challis *et al.*, 2020
Hifiasm	0.16.1-r375	Cheng *et al.*, 2021
HiGlass	1.11.6	Kerpedjiev *et al.*, 2018
MitoHiFi	2.0	Uliano-Silva *et al.*, 2021
PretextView	0.2.5	https://github.com/wtsi- hpag/PretextView
purge_dups	1.2.3	Guan *et al.*, 2020
YaHS	1.1.91eebc2	Zhou *et al.*, 2022

### Genome annotation

The Ensembl gene annotation system ( Aken *et al.*, 2016) was used to generate annotation for the *T. femorata* assembly (GCA_944319695.1). Annotation was created primarily through alignment of transcriptomic data to the genome, with gap filling via protein to-genome alignments of a select set of proteins from UniProt ( UniProt Consortium, 2019).

### Ethics/compliance issues

The materials that have contributed to this genome note have been supplied by a Darwin Tree of Life Partner. The submission of materials by a Darwin Tree of Life Partner is subject to the Darwin Tree of Life Project Sampling Code of Practice. By agreeing with and signing up to the Sampling Code of Practice, the Darwin Tree of Life Partner agrees they will meet the legal and ethical requirements and standards set out within this document in respect of all samples acquired for, and supplied to, the Darwin Tree of Life Project. Each transfer of samples is further undertaken according to a Research Collaboration Agreement or Material Transfer Agreement entered into by the Darwin Tree of Life Partner, Genome Research Limited (operating as the Wellcome Sanger Institute), and in some circumstances other Darwin Tree of Life collaborators.

## Data Availability

European Nucleotide Archive: *Tiphia femorata* (beetle killing wasp). Accession number PRJEB51917; https://identifiers.org/ena.embl/PRJEB51917 ( Wellcome Sanger Institute, 2022). The genome sequence is released openly for reuse. The *T. femorata* genome sequencing initiative is part of the Darwin Tree of Life (DToL) project. All raw sequence data and the assembly have been deposited in INSDC databases. The genome will be annotated and presented through the Ensembl pipeline at the European Bioinformatics Institute. Raw data and assembly accession identifiers are reported in Table 1.

## Author information

Members of the University of Oxford and Wytham Woods Genome Acquisition Lab are listed here: https://doi.org/10.5281/zenodo.4789928.

Members of the Darwin Tree of Life Barcoding collective are listed here: https://doi.org/10.5281/zenodo.4893703.

Members of the Wellcome Sanger Institute Tree of Life programme are listed here: https://doi.org/10.5281/zenodo.4783585.

Members of Wellcome Sanger Institute Scientific Operations: DNA Pipelines collective are listed here: https://doi.org/10.5281/zenodo.4790455.

Members of the Tree of Life Core Informatics collective are listed here: https://doi.org/10.5281/zenodo.5013541.

Members of the Darwin Tree of Life Consortium are listed here: https://doi.org/10.5281/zenodo.4783558.
