# Supplementary material for: The genome sequence of a beetle-killing wasp, Tiphia femorata (Fabricius, 1775)
Source: Wellcome Open Res. 2023 Feb 3;8:57. [Version 1] doi: 10.12688/wellcomeopenres.18893.1 (PMC10687388; doi:10.12688/wellcomeopenres.18893.1)
Supplement: Peer review history

# Reviewer report: Reviewer response for version 1

**DOI:** 10.21956/wellcomeopenres.20949.r67957

**Published:** 2023-11-29

**Authors:** Željko Tomanović

**Affiliations:** 1 Serbia University of Belgrade, Belgrade, Serbia

The authors presented the genome assembly of *Tiphia femorata,* a beetle-killing wasp whose larvae prey on various beetle larvae. They found 10,470 protein-coding genes. They also assembled the complete mitochondrial genome. This paper is worth publishing. I have just a few minor suggestions.

*Tiphia femorata*, the wasp sequenced here, probably represents a species complex (see FaunaEuropea - https://fauna-eu.org/). Could the authors clarify the identification issue? Also, the common host *Rhizotrogus solstitialis* is a synonym of *Amphimallon solstitiale*, so *A. solstitiale* is the correct species name. Please correct.

Are sufficient details of methods and materials provided to allow replication by others?

Yes

Is the rationale for creating the dataset(s) clearly described?

Yes

Are the datasets clearly presented in a useable and accessible format?

Yes

Are the protocols appropriate and is the work technically sound?

Yes

Reviewer Expertise:

diversity and ecology of parasitoid wasps

I confirm that I have read this submission and believe that I have an appropriate level of expertise to confirm that it is of an acceptable scientific standard.

# Reviewer report: Reviewer response for version 1

**DOI:** 10.21956/wellcomeopenres.20949.r67952

**Published:** 2023-10-10

**Authors:** Feng Zhang

**Affiliations:** 1 Nanjing Agricultural University, Nanjing, China

The authors present a genome assembly from an individual female *Tiphia femorata* by combining HiFi long reads and HI-C data. This chromosomal-level assembly spans approximately 276 Mb with 98.73% of the assembly being scaffolded into 12 chromosomal pseudomolecules.

 As a result, it produces a high-quality genome assembly. Furthermore, gene annotation performed on this assembly using Ensembl has identified 10,470 protein-coding genes.

 In the Genome sequence report, the authors mentioned that the deposited assembly represents one haplotype. However, they also indicated that contigs corresponding to the second haplotype have been deposited. While the accession of the alternate haplotype is provided, it would be beneficial to include specific assembly information, such as the genome size and scaffold N50.

 Minor revisions:

 In Background, change “is an abundant species in most of Europe” to “is an abundant species in most areas of Europe”.

Are sufficient details of methods and materials provided to allow replication by others?

Yes

Is the rationale for creating the dataset(s) clearly described?

Yes

Are the datasets clearly presented in a useable and accessible format?

Yes

Are the protocols appropriate and is the work technically sound?

Yes

Reviewer Expertise:

Insect genomics, phylogenomics

I confirm that I have read this submission and believe that I have an appropriate level of expertise to confirm that it is of an acceptable scientific standard.
